# Facteurs prédictifs de la réponse aux biothérapies chez les patients atteints de polyarthrite rhumatoïde: étude observationnelle transversale menée au Service de Rhumatologie de l’Hôpital Militaire d’Instruction Mohammed V, Rabat (Maroc)

**DOI:** 10.11604/pamj.2025.52.172.47745

**Published:** 2025-12-19

**Authors:** Mahamadou Sagara, Abderrahim Majjad, Hamza Toufik, Ahmed Bezza

**Affiliations:** 1 Service de Rhumatologie, Hôpital Militaire d'Instruction Mohammed V, Rabat, Maroc,; 2 Service de Rhumatologie, Faculté de Médecine, de Pharmacie de Rabat, Hôpital Militaire d'Instruction Mohammed V, Rabat, Maroc

**Keywords:** Rheumatoid arthritis, biologic therapy, clinical response, comorbidities, biomarkers, diabetes, C-reactive protein (CRP), Polyarthrite rhumatoïde, biothérapie, réponse clinique, comorbidités, biomarqueurs, diabète, protéine C réactive

## Abstract

**Introduction:**

la réponse thérapeutique aux biothérapies dans la polyarthrite rhumatoïde (PR) demeure hétérogène, influencée par divers facteurs cliniques, biologiques et comorbidités. L'objectif de cette étude était d'identifier les facteurs associés à une bonne réponse clinique aux biothérapies chez des patients atteints de PR suivis dans un contexte nord-africain.

**Méthodes:**

une étude observationnelle transversale a été menée au Service de Rhumatologie de l'Hôpital Militaire d'Instruction Mohammed V de Rabat entre janvier et décembre 2024. Les données sociodémographiques, cliniques, biologiques et de comorbidité des patients traités par biothérapie ont été recueillies au moment de leur suivi. Une régression logistique multivariée a été réalisée afin d'identifier les facteurs indépendamment associés à une bonne réponse clinique selon les critères de l'European League Against Rheumatism (EULAR).

**Résultats:**

cent cinquante-trois patients ont été inclus, dont 75,8% de femmes (âge moyen: 57,5 ± 13,7 ans). En analyse multivariée, la positivité des anticorps anti-CCP (ORa = 3,15; IC95%: 1,62-6,14; P = 0,001) et un délai d'instauration de la biothérapie ≤ 1 an (ORa = 4,55; IC95%: 2,12-9,76; P < 0,001) étaient significativement associés à une meilleure réponse clinique. À l'inverse, une CRP > 15 mg/L (ORa = 0,29; IC95%: 0,14-0,63; P < 0,001) et la présence d'un diabète (ORa = 0,33; IC95%: 0,15-0,74; P = 0,003) étaient associées à une réponse défavorable.

**Conclusion:**

la positivité des anticorps anti-CCP et l'instauration précoce d'une biothérapie sont associées à une meilleure réponse clinique, tandis qu'un taux élevé de CRP et la présence d'un diabète sont liés à une évolution moins favorable. Ces résultats soulignent la nécessité d'une prise en charge précoce et individualisée de la PR, en particulier dans les contextes à ressources limitées.

## Abstract (en)

**Introduction:**

therapeutic response to biologic agents in rheumatoid arthritis (RA) remains heterogeneous and is influenced by clinical, biological, and comorbid factors. This study aimed to identify the factors associated with a good clinical response to biologic therapy among RA patients followed in a North African context.

**Methods:**

a cross-sectional observational study was conducted at the Rheumatology Department of Mohammed V Military Teaching Hospital, Rabat, between January and December 2024. Sociodemographic, clinical, biological, and comorbidity data of patients treated with biologic agents were collected at the time of follow-up. Multivariable logistic regression analysis was performed to identify independent factors associated with a good clinical response according to the European League Against Rheumatism (EULAR) criteria.

**Results:**

a total of 153 patients were included, of whom 75.8% were women (mean age 57.5 ± 13.7 years). In multivariable analysis, anti-CCP antibody positivity (aOR = 3.15; 95% CI 1.62-6.14; P = 0.001) and initiation of biologic therapy within 1 year of diagnosis (aOR = 4.55; 95% CI 2.12-9.76; P < 0.001) were significantly associated with a better clinical response. Conversely, C-reactive protein > 15 mg/L (aOR = 0.29; 95% CI 0.14-0.63; P < 0.001) and diabetes (aOR = 0.33; 95% CI 0.15-0.74; P = 0.003) were associated with a poorer response.

**Conclusion:**

anti-CCP antibody positivity and early initiation of biologic therapy were associated with a better clinical response, whereas elevated CRP levels and diabetes were linked to an unfavourable therapeutic outcome. These findings highlight the importance of early and individualised management of RA, particularly in resource-limited settings.

## Introduction

La polyarthrite rhumatoïde (PR) est une maladie auto-immune chronique caractérisée par une inflammation persistante des articulations, entraînant des destructions structurales progressives et un handicap fonctionnel majeur. L'introduction des biothérapies ciblant les voies immuno-inflammatoires, telles que les anti-TNF, les anti-IL-6, les anti-CD20 ou les inhibiteurs de JAK, a profondément modifié le pronostic fonctionnel et structural de la PR au cours des deux dernières décennies [1-3]. Cependant, la réponse clinique à ces traitements reste hétérogène : environ un tiers des patients ne présentent pas d'amélioration significative selon les critères EULAR à six mois [4,5]. Cette variabilité s'explique par des différences interindividuelles liées à la génétique, à l'immunopathologie, aux comorbidités métaboliques et au moment d'instauration du traitement [6-8].

Plusieurs études ont identifié des biomarqueurs sérologiques tels que les anticorps anti-CCP (ACPA) ou le facteur rhumatoïde (FR) comme prédicteurs de bonne réponse, tandis qu'une inflammation systémique élevée ou la présence de comorbidités métaboliques, notamment le diabète, a été associée à une moindre efficacité [9-11]. Néanmoins, la majorité de ces travaux provient de cohortes européennes et nord-américaines, dont les caractéristiques sociodémographiques et l'accès aux soins diffèrent de ceux observés dans les pays à ressources limitées. Au Maroc, les données locales sur les facteurs prédictifs de réponse aux biothérapies dans la PR demeurent rares. L'accès différé à ces traitements, souvent conditionné par des contraintes économiques et organisationnelles, pourrait influencer les taux de réponse observés. Ainsi, l'objectif de cette étude était d'identifier, dans une cohorte marocaine de patients atteints de PR, les facteurs cliniques, biologiques et les comorbidités associés à la réponse clinique aux biothérapies à six mois selon les critères EULAR.

## Méthodes

**Type d'étude et cadre:** il s'agit d'une étude observationnelle transversale menée en pratique réelle au sein du Service de Rhumatologie de l'Hôpital Militaire d'Instruction Mohammed V de Rabat (Maroc), centre national de référence pour les maladies rhumatologiques inflammatoires. La collecte des données a été réalisée entre le 1^er^ janvier et le 31 décembre 2024 auprès des patients consultant pour un suivi de polyarthrite rhumatoïde (PR) et recevant une biothérapie au moment de l'enquête.

**Population d'étude:** la population étudiée comprenait tous les patients adultes (≥ 18 ans) atteints de polyarthrite rhumatoïde (PR) diagnostiquée selon les critères de classification ACR/EULAR 2010, sous traitement par biothérapie et ayant un suivi clinique documenté au moment de la collecte. Ont été exclus les patients présentant une autre arthropathie inflammatoire, une maladie auto-immune associée, un antécédent de cancer actif ou un dossier médical incomplet. L'échantillon final comptait 153 patients correspondant à l'ensemble des sujets éligibles suivis dans le service durant la période considérée. Aucun calcul d'effectif préalable n'a été réalisé, le recrutement ayant porté sur la totalité des patients répondant aux critères d'inclusion.

**Collecte des données:** les données ont été recueillies de manière transversale à partir des consultations et bilans cliniques effectués durant la période d'étude, à l'aide d'une fiche standardisée. Les variables collectées comprenaient: i) données démographiques: âge, sexe, indice de masse corporelle (IMC), niveau d'instruction; ii) données cliniques: durée d'évolution de la PR, délai entre le diagnostic et l'instauration de la biothérapie, scores d'activité (DAS28-CRP, SDAI, CDAI), score HAQ, EVA douleur, présence d'érosions radiographiques; iii) données biologiques: taux de CRP, facteur rhumatoïde (FR), anticorps anti-CCP (ACPA); iv) comorbidités: hypertension artérielle, diabète (HbA1c ≥ 6,5% ou traitement hypoglycémiant), dyslipidémie, tabagisme, cardiopathie, dépression, fibromyalgie; v) traitements: biologiques (anti-TNF, rituximab, tocilizumab, inhibiteurs de JAK).

**Définitions:** la variable principale étudiée était la réponse clinique à la biothérapie, évaluée selon les critères de *l'European League Against Rheumatism (EULAR)* basés sur le score DAS28-CRP. Selon ces critères: i) un patient est classé « bon répondeur » *(good responder)* s'il présente une diminution du DAS28-CRP > 1,2 associée à une valeur finale ≤ 3,2 (activité faible ou rémission); ii) un patient est classé « répondeur modéré » *(moderate responder)* si la diminution du DAS28-CRP est comprise entre 0,6 et 1,2 ou si la valeur finale reste > 3,2 mais ≤ 5,1 (activité modérée); un patient est classé « non-répondeur » *(non-responder)* si la diminution du DAS28-CRP ≤ 0,6, ou si la valeur finale reste > 5,1, traduisant une activité persistante élevée. Pour les besoins analytiques de cette étude, les bons et modérés répondeurs ont été regroupés dans la catégorie « répondeurs », tandis que les autres ont été classés comme « non-répondeurs ». Une CRP élevée a été définie par une valeur strictement supérieure à 15 mg/L, seuil choisi pour refléter une activité inflammatoire systémique cliniquement significative. Les scores d'activité de la maladie DAS28-CRP *(Disease Activity Score)*, SDAI *(Simplified Disease Activity Index)* et CDAI *(Clinical Disease Activity Index)* ont été calculés selon les formules standardisées de l'*EULAR*, intégrant: i) le nombre d'articulations sensibles et gonflées (sur 28); ii) l'évaluation globale du patient (EVA, 0-100 mm); iii) la valeur de la CRP (mg/L).

**Analyse statistique:** les analyses ont été réalisées à l'aide du logiciel JAMOVI v26.6. Les variables quantitatives ont été décrites en moyenne ± écart-type ou en médiane [IQR] selon leur distribution (test de Shapiro-Wilk) et comparées à l'aide du test t de Student ou de l'ANOVA. Les variables qualitatives ont été comparées par le test du X^2^ ou le test exact de Fisher. Une analyse bivariée a d'abord été effectuée pour identifier les variables associées à la réponse clinique (P < 0,20). Les variables significatives ont ensuite été incluses dans un modèle de régression logistique multivariée, ajusté sur l'âge, le sexe et la présence d'érosions radiographiques. Les résultats sont présentés sous forme d'odds ratios ajustés (ORa), avec leurs intervalles de confiance à 95% (IC95%) et leurs valeurs de P exactes (P = 0,04, P < 0,001, etc.). Le seuil de significativité a été fixé à P < 0,05.

**Considérations éthiques:** l'approbation éthique de cette étude a été obtenue auprès du comité d'éthique de l'Hôpital Militaire d'Instruction Mohammed V de Rabat (Maroc). Le numéro de référence officiel est en cours d'attribution et sera communiqué dès réception. Tous les participants ont donné un consentement éclairé écrit, conformément à la déclaration d'Helsinki. Les données ont été traitées de manière strictement confidentielle et anonymisées avant analyse.

## Résultats

**Figure 1:**
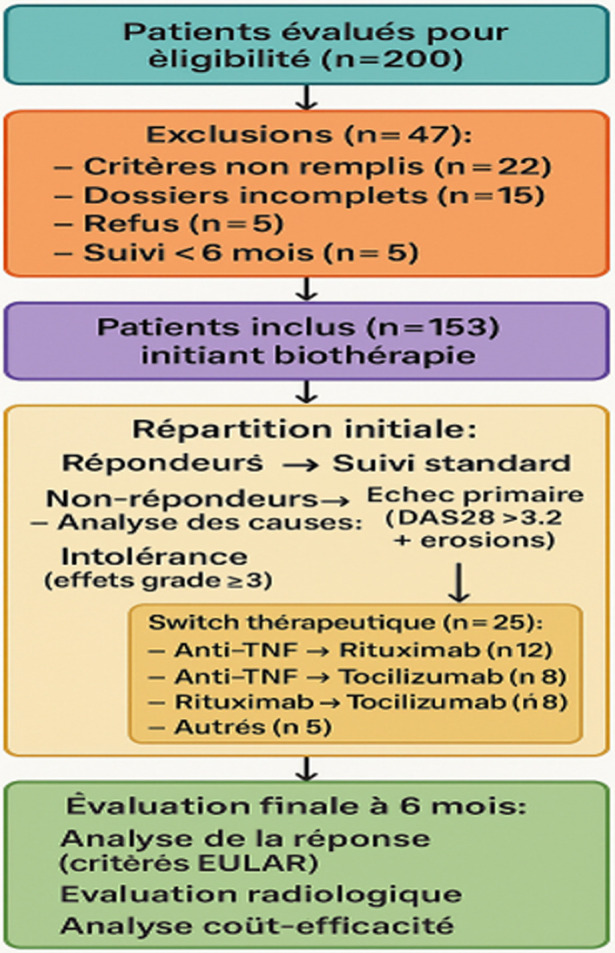
diagramme de flux des patients inclus dans l'étude

**Participants:** parmi les 200 patients initialement évalués pour l'éligibilité, 47 ont été exclus: 22 ne remplissaient pas les critères d'inclusion, 15 présentaient des dossiers incomplets, 5 ont refusé de participer et 5 avaient un suivi inférieur à six mois. Au total, 153 patients ont été inclus et ont initié une biothérapie, avec un suivi complet jusqu'à six mois. Aucun patient n'a été perdu de vue ni décédé durant la période d'observation. Vingt-cinq patients (16,3%) ont nécessité un changement de biothérapie, principalement pour échec primaire (64%) ou effets indésirables sévères (28%), dont trois infections graves. Le processus de sélection, d'inclusion et de suivi des participants est illustré dans la Figure 1.

**Caractéristiques de la population:** les caractéristiques sociodémographiques, cliniques et biologiques des patients sont résumées dans le Tableau 1. L'âge moyen était de 57,5 ± 13,7 ans, avec une prédominance féminine (75,8%). La durée moyenne d'évolution de la polyarthrite rhumatoïde (PR) était de 15,2 ± 9,3 ans et le délai médian entre le diagnostic et la première biothérapie était de 8,1 ± 7,8 ans. À l'inclusion, le score moyen DAS28-CRP était de 3,79 ± 1,78, traduisant une activité modérée à élevée; 52,3% des patients présentaient une CRP > 15 mg/L. Le score HAQ moyen était de 0,97 ± 0,69. Les comorbidités les plus fréquentes étaient l'hypertension artérielle (28,8%), le diabète (14,4%), la dyslipidémie (16,7%) et l'obésité (21,1%). Des érosions radiographiques étaient présentes chez 64,4% des patients. Les données principales utilisées pour les analyses statistiques étaient complètes (< 2% de valeurs manquantes pour le SDAI et le CDAI).

**Tableau 1:** caractéristiques cliniques, biologiques et comorbidités à l'inclusion (n = 153)

Paramètre	Valeur
Âge (ans), moyenne ± ET	57,5 ± 13,7
Sexe féminin, n (%)	116 (75,8 %)
Durée de la PR (années), moyenne ± ET	15,2 ± 9,3
Délai diagnostic → biothérapie (années)	8,1 ± 7,8
DAS28-CRP initial	3,79 ± 1,78
CDAI / SDAI moyens	15,9 / 17,8
CRP (mg/L), moyenne ± ET	19,3 ± 29,3
HAQ / EVA douleur (0–10)	0,97 ± 0,69 / 4,53 ± 2,42
IMC (kg/m^2^), moyenne ± ET	26,1 ± 4,3
**Comorbidités (%)**	-
• Diabète	14,4
• HTA	28,8
• Dyslipidémie	16,7
• Cardiopathie	10,5
• Dépression	10,6
• Fibromyalgie	12,4
• Tabagisme actif	3,9
FDR cardiovasculaires globaux	53,3
Érosions radiologiques	64,4
CRP > 15 mg/L	52,3


DAS28: disease activity score; CRP: C-reactive protein; CDAI: clinical disease activity index; SDAI: simplified disease activity index; HAQ: health assessment questionnaire; EVA: échelle visuelle analogique; IMC: indice de masse corporelle; HTA: hypertension artérielle; FDR: facteur de risque

**Réponse clinique à six mois:** après six mois de traitement, 92 patients (60,1%) présentaient une bonne réponse clinique selon les critères EULAR. Le taux de changement de biothérapie était de 16,3%, principalement pour échec primaire, avec un délai médian de switch de 14 semaines [IQR: 8-20]. La proportion de bons répondeurs variait selon les classes thérapeutiques sans différence significative (P > 0,05): 62,4% pour les anti-TNF, 58,7% pour le rituximab, 60,5% pour le tocilizumab et 60% pour les inhibiteurs de JAK (Tableau 2).

**Tableau 2:** réponse clinique à six mois selon les classes de biothérapies et le changement de traitement (n = 153)

Paramètres	Effectif (n, %)	Commentaires / Détails
**Réponse clinique globale à 6 mois (critères EULAR)**	92 (60,1%) : bonne réponse 61 (39,9 %) : réponse insuffisante	-
**Changement de biothérapie avant 6 mois**	25 (16,3 %)	Délai médian : 14 semaines [IQR 8–20]
**Échec primaire**	16 (64,0%)	DAS28 > 3,2 à 3 mois
**Échec secondaire**	4 (16,0%)	Perte d’efficacité
**Intolérance / effets indésirables graves**	7 (28,0%)	Dont 3 infections sévères
**Réponse EULAR selon la classe de biothérapie**		-
**Anti-TNF (n = 93)**	58 (62,4%) bonne réponse / 35 (37,6%) insuffisante	P = .42
**Rituximab (n = 92)**	54 (58,7%) / 38 (41,3%)	P = .65
**Tocilizumab (n = 38)**	23 (60,5%) / 15 (39,5%)	P = .72
**Inhibiteurs de JAK (n = 5)**	3 (60,0%) / 2 (40,0%)	P = .99

EULAR: European League Against Rheumatism; TNF: tumor necrosis factor; JAK: Janus Kinase; IQR: interquartile range

**Analyse bivariée et multivariée:** en analyse bivariée (Tableau 3), les répondeurs (n = 92) présentaient un score DAS28-CRP initial significativement plus bas que les non-répondeurs (3,5 ± 1,6 vs 4,2 ± 1,9 ; P = 0,02), une CRP ≤ 15 mg/L plus fréquente (62% vs 26,2%; P < 0,001), une positivité ACPA plus élevée (75% vs 45,9%; P < 0,001) et une prévalence plus faible du diabète (14,1% vs 34,4%; P = 0,003). Aucune différence significative n'a été observée selon l'âge, le sexe ou la présence d'hypertension artérielle (P > 0,05). En analyse multivariée (Tableau 3), la positivité des anticorps anti-CCP demeurait un facteur indépendant de bonne réponse clinique (ORa = 3,15; IC95%: 1,62-6,14; P = 0,001) ; un délai diagnostic-biothérapie ≤ 1 an était également associé à une meilleure réponse (ORa = 4,55; IC95%: 2,13-9,72; P < 0,001). À l'inverse, une CRP > 15 mg/L (ORa = 0,29; IC95%: 0,14-0,63; P = 0,001) et la présence d'un diabète (ORa = 0,33; IC95%: 0,15-0,72; P = 0,003) étaient associées à une moindre probabilité de réponse favorable. Le modèle ajusté sur l'âge, le sexe et la présence d'érosions radiographiques expliquait 41% de la variance (pseudo-R^2^ = 0,41), traduisant une bonne qualité d'ajustement.

**Tableau 3:** facteurs associés à la réponse clinique selon les critères EULAR à six mois (analyses bivariées et multivariées ajustées sur l'âge, le sexe et les érosions radiographiques)

Facteurs	Répondeurs (n = 92)	Non-répondeurs (n = 61)	Analyse bivariée (P)	Analyse multivariée ORa (IC95%)	P
Âge (ans), moyenne ± ET	56,2 ± 12,8	59,1 ± 14,5	0,18	-	-
Sexe féminin, %	74,4	78,7	0,52	-	-
DAS28-CRP initial, moyenne ± ET	3,5 ± 1,6	4,2 ± 1,9	0,02	-	-
ACPA positifs, %	75,0	45,9	< 0,001	3,15 (1,62 - 6,14)	0,001
CRP > 15 mg/L, %	38,0	73,8	< 0,001	0,29 (0,14 - 0,63)	0,001
Diabète, %	14,1	34,4	0,003	0,33 (0,15 - 0,72)	0,003
Érosions radiographiques, %	58,7	75,4	0,03	-	-
Délai diagnostic → biothérapie ≤ 1 an, %	29,3	9,8	< 0,001	4,55 (2,13 - 9,72)	< 0,001


EULAR: European League Against Rheumatism; ORa: odds ratio ajusté; IC95%: intervalle de confiance à 95%; ACPA: anticorps anti-peptides citrullinés; CRP: protéine C-réactive; DAS28: disease activity score sur 28 articulations; ET: écart-type

**Figure 2:**
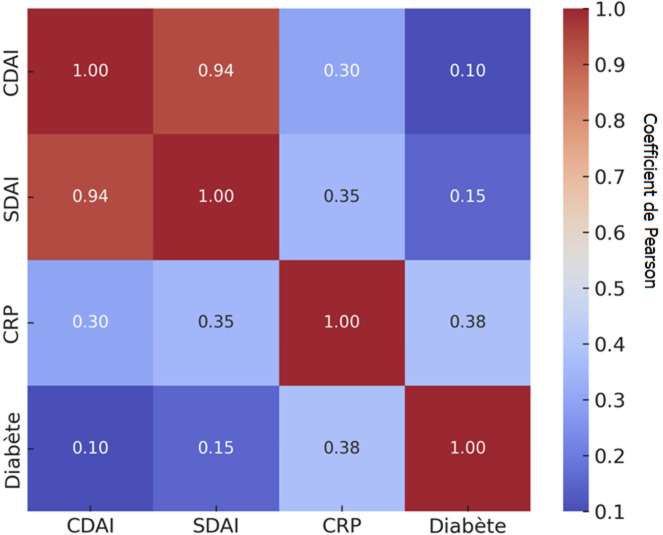
corrélations cliniques et biologiques (coefficients de Pearson)

**Figure 3:**
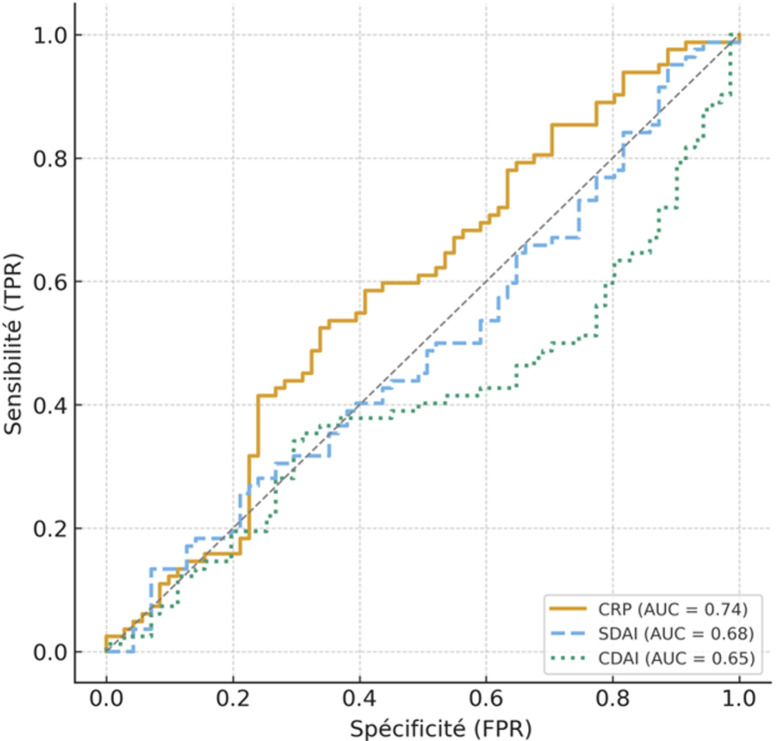
performance prédictive des marqueurs CRP, SDAI et CDAI dans la réponse thérapeutique (courbes ROC)

**Analyses complémentaires:** les corrélations (Figure 2) montraient des associations significatives entre le CDAI et le SDAI (r = 0,94; P < 0,001, la CRP et les érosions (r = 0,42; P = 0,008), ainsi que la CRP et le diabète (r = 0,38; P = 0,02), suggérant un lien entre inflammation systémique, atteinte structurale et comorbidités métaboliques. L'analyse des courbes ROC (Figure 3) a identifié la CRP comme le meilleur marqueur prédictif de réponse thérapeutique (AUC = 0,74 ; IC95%: 0,66-0,81; P < 0,001), suivie du SDAI (AUC = 0,68; IC95% : 0,60-0,76; P = 0,004) et du CDAI (AUC = 0,65; IC95%: 0,56-0,74; P = 0,008).

## Discussion

Cette étude observationnelle transversale menée sur une cohorte marocaine de 153 patients atteints de polyarthrite rhumatoïde (PR) avait pour objectif d'identifier les facteurs cliniques, biologiques et comorbidités associés à la réponse clinique aux biothérapies selon les critères de l'EULAR. Les résultats ont montré que la positivité des anticorps anti-CCP (ACPA) était significativement associée à une meilleure réponse clinique (ORa = 3,15 ; IC95%: 1,62-6,14 ; P = 0,001), tout comme un délai court (≤ 1 an) entre le diagnostic et l'instauration du traitement biologique (ORa = 4,55; IC95%: 2,13-9,72; P < 0,001). À l'inverse, une CRP > 15 mg/L (ORa = 0,29; IC95%: 0,14-0,63; P = 0,001) et la présence d'un diabète (ORa = 0,33; IC95%: 0,15-0,72; P = 0,003) étaient associées à une réponse défavorable. Aucune différence d'efficacité n'a été observée entre les différentes classes de biothérapies (anti-TNF, anti-IL6, anti-CD20, inhibiteurs de JAK; P > 0,05).

Ces résultats confirment la variabilité interindividuelle de la réponse clinique rapportée dans les grandes cohortes internationales. L'identification du diabète comme facteur associé à une moindre réponse constitue un apport original de cette étude. Cette relation pourrait refléter une interaction délétère entre inflammation métabolique et auto-immunité, altérant la biodisponibilité des agents biologiques et la sensibilité des voies de signalisation des cytokines [12]. Des travaux européens et nord-américains ont également montré que la positivité des ACPA constitue un marqueur robuste de meilleure réponse, notamment aux anti-TNF et au rituximab [13-15]. L'association entre un délai court d'instauration de la biothérapie et une meilleure réponse soutient le concept de « fenêtre thérapeutique précoce », décrit dans les études BeSt et TEAR [16-18]. Inversement, une inflammation systémique persistante, mise en évidence par une CRP élevée, semble réduire la probabilité d'une réponse optimale aux biothérapies [19,20].

Dans les pays à ressources limitées, où le contrôle des comorbidités métaboliques comme le diabète demeure souvent imparfait, cette interaction pourrait expliquer en partie la variabilité de la réponse clinique observée. L'absence de différence significative entre les classes thérapeutiques rejoint les conclusions de registres tels que Biobadaser et Rabbit [21], suggérant une efficacité comparable en pratique réelle. Toutefois, ces résultats doivent être interprétés avec prudence, le choix de la biothérapie étant fréquemment influencé par des contraintes économiques. Le rituximab étant privilégié en première ligne dans 57% des cas. La corrélation entre CRP, érosions radiographiques et diabète pourrait traduire un phénotype inflammatoire-métabolique particulier, moins répondeur aux biothérapies conventionnelles et encore insuffisamment exploré [22].

Sur le plan clinique, ces résultats soulignent l'importance d'un diagnostic précoce, d'une initiation rapide des traitements ciblés et d'une prise en charge intégrée des comorbidités métaboliques, en particulier du diabète. L'intégration de ces paramètres dans la stratégie thérapeutique pourrait favoriser une médecine plus personnalisée et une allocation rationnelle des ressources dans les contextes à ressources limitées. Malgré sa rigueur méthodologique, cette étude présente certaines limites. La taille de l'échantillon (n = 153) réduit la puissance statistique des comparaisons intergroupes, notamment pour les inhibiteurs de JAK (n = 5). Le caractère observationnel et transversal ne permet pas d'établir une relation causale entre les variables étudiées. De plus, certains facteurs confondants, tels que l'adhésion thérapeutique, le statut socio-économique ou les disparités d'accès aux soins, n'ont pu être pleinement pris en compte. L'absence de biomarqueurs immunologiques avancés (IgA-RF, ACPA multi-épitope, interleukines) limite également l'exploration des mécanismes sous-jacents. Enfin, la validité externe est limitée au contexte marocain, marqué par un délai médian de mise en place prolongé des biothérapies et par des contraintes économiques significatives, ce qui pourrait influer sur la généralisabilité des résultats.

## Conclusion

Cette étude menée au sein d'une cohorte marocaine de patients atteints de polyarthrite rhumatoïde a permis d'identifier plusieurs facteurs déterminants de la réponse clinique aux biothérapies à six mois. La positivité des anticorps anti-CCP et l'instauration précoce du traitement biologique (≤ 1 an après le diagnostic) étaient associées à une meilleure probabilité de réponse, tandis qu'une CRP élevée (> 15 mg/L) et la présence d'un diabète constituaient des facteurs indépendants de non-réponse. Ces résultats confirment l'importance de la précocité thérapeutique et du contrôle des comorbidités métaboliques dans l'optimisation de la réponse clinique. Ils soulignent également la nécessité d'une approche intégrée et personnalisée de la prise en charge de la PR, particulièrement dans les contextes à ressources limitées.

### Etat des connaissances sur le sujet

- La réponse clinique aux biothérapies dans la polyarthrite rhumatoïde est variable et influencée par des facteurs immunologiques, inflammatoires et des comorbidités;
- Les anticorps anti-CCP sont associés à une meilleure réponse aux biothérapies, en particulier aux anti-TNF et au rituximab;
- Une inflammation systémique élevée, mesurée par la CRP, et la présence de comorbidités métaboliques réduisent l'efficacité thérapeutique.

### Contribution de notre étude à la connaissance

- Le diabète est identifié comme facteur indépendant de non-réponse clinique aux biothérapies dans une cohorte marocaine de polyarthrite rhumatoïde;
- Un délai diagnostic-traitement ≤ 1 an augmente significativement la probabilité de réponse clinique favorable;
- Ces résultats soutiennent la mise en œuvre précoce et personnalisée des biothérapies dans les contextes à ressources limitées.

## Remerciements

Les auteurs remercient chaleureusement le personnel paramédical du Service de Rhumatologie de l'Hôpital Militaire d'Instruction Mohammed V de Rabat pour son aide précieuse dans la collecte des données et le suivi des patients. Ils expriment également leur gratitude au Département de Pharmacie Hospitalière pour l'accès aux données de prescription, ainsi qu'aux équipes de radiologie pour leur participation à l'évaluation des lésions structurales. Aucun rédacteur médical professionnel n'a été sollicité.
